# Short-term effects of sweetened acidic beverages consumption on human saliva: Colloidal properties and protein composition

**DOI:** 10.1371/journal.pone.0330023

**Published:** 2025-09-03

**Authors:** Samina Zaheer, Ben Kew, Chris Louca, Sassan Hafizi, Anwesha Sarkar, Mahdi Mutahar

**Affiliations:** 1 School of Dental, Health and Care Professions, University of Portsmouth, Portsmouth, United Kingdom; 2 Food Colloids and Bioprocessing Group, School of Food Science and Nutrition, University of Leeds, Leeds, United Kingdom; 3 Cell and Molecular Biology department, Faculty of Science & Health, School of Medicine, Pharmacy and Biomedical Sciences, University of Portsmouth, Portsmouth, United Kingdom; University of Pennsylvania, UNITED STATES OF AMERICA

## Abstract

This study investigates the impact of a sweetened acidic beverage, an apple juice (J) consumption on the tribological properties, viscoelasticity, and protein concentration/ composition of human saliva. Using a combination of tribological measurements, quartz crystal microbalance with dissipation monitoring (QCM-D), and protein analysis, we assessed how J may affect saliva’s lubricating behaviour and adsorbed salivary film characteristics compared to water (control). Tribological results revealed that saliva (collected from 32 healthy adults) exposed to water or J exhibited increased friction when compared to unstimulated whole mouth saliva (uWMS), particularly within the boundary lubrication regime. A one-min rinse with water or J caused salivary delubrication, with water having a greater delubricating effect (*p* *<* *0.05*) than that of J. Strikingly, the friction coefficients reverted to those for uWMS after 10 min (*p* *>* *0.05*) highlighting the transient nature of delubrication caused by J consumption. This transient phenomenon was also evident in QCM-D measurements, where J transformed the ex vivo salivary film into a rigid layer, which reverted upon buffer application on model hydrophilic surfaces. Analysis of total protein concentration (TPC) showed that water significantly reduced TPC after one min, while J required 10 min to achieve similar TPC reduction. Sodium dodecyl sulphate polyacrylamide gel electrophoresis (SDS-PAGE) revealed that salivary cystatins and carbonic anhydrase significantly changed after J intervention, unlike water. This study highlights limited effects of J on salivary delubrication and adsorption. Building upon our findings, future research should investigate how repeated exposure to sweetened acidic beverages influences *in vivo* salivary pellicle dynamics and impacts to oral health.

## 1. Introduction

Human saliva plays a crucial role in dental homeostasis, thanks to its viscoelastic flow behaviour and protein composition [1]. Saliva contains over 1000 proteins, including mucin, statherin, cystatins, proline-rich proteins (PRPs), and Secretory immunoglobulin A (SIgA), which are essential for lubrication [2,3]. Oral bio-lubrication is considered to be primarily provided by adsorbed layers of salivary proteins such as mucin, statherin, proline-rich proteins, and also other small molecular proteins [4–6]. Anionic glycosylated mucins at oral pH, particularly MUC5B, a large glycoprotein enriched with hydrophilic carbohydrate side chains, and statherins, provide saliva with its unique viscoelastic, water-retaining, and to a certain degree its lubricating properties [4,7–10]. It is, therefore, important to consider the influence of saliva and mucosal salivary pellicle in the lubrication of oral surfaces by saliva, a thin proteinaceous layer formed by the adsorption of salivary proteins onto the teeth and oral epithelium, which plays a crucial role in reducing friction and lubricate, thus protecting both hard and soft oral tissues from bacterial attack, fungal growth, and de-mineralization of teeth [11,12]. The oral cavity contains both hydrophobic surfaces, such as the soft tissues (e.g., the buccal mucosa), and hydrophilic surfaces, such as teeth, which interact differently with saliva and influence lubrication and protein adsorption dynamics. Previous studies have shown that the lubricious boundary film-forming nature of salivary proteins is largely attributed to its protein and type of substrate, further demonstrating their role in reducing friction [9,13,14]. This helps prevent damage from mechanical forces such as mastication and speech, the erosive potential of drinks and foods as well as providing a defence against pathogenic invasion [15–17]. Unstimulated saliva, characterized by its higher protein content and viscosity, may account for the observed friction differences compared to stimulated saliva [18].

Literature has shown that the consumption of acidic beverages such as iced tea and fizzy colas significantly increases the flow rate of whole mouth saliva (WMS) and enhances its elasticity. In contrast, water-stimulated saliva has been found to exhibit lower elasticity [19]. Dietary acids, particularly from carbonated drinks and fruit juices, are widely consumed globally, with some countries even counting a serving of fruit juice as part of daily fruit intake [20]. However, despite its nutritional appeal, apple juice contains natural sugars and dietary acids that may negatively affect oral health by altering salivary composition, impairing lubrication, and contributing to enamel erosion, particularly with frequent or prolonged exposure. Given its popularity and perceived benefits, a deeper understanding of how apple juice interacts with the oral environment is both timely and necessary. Their frequent consumption, especially between meals rather than during meals, is strongly linked to adverse effects on the oral environment, especially on tooth enamel [21,22]. Fruit juices containing acids such as citric, malic, and tartaric acids, along with sugars like sucrose, glucose, fructose, and sorbitol, to make these drinks more consumable and palatable, cause significant damage to teeth [21–24]. Studies have shown that the salivary pH drops immediately after consuming fruit drinks, with an oral clearance rate of about 15 minutes, leading to a significant cariogenic and erosive tooth wear potential [25,26]. Fruit juices require higher alkaline stimulated saliva to be neutralised than carbonated drinks due to their higher sugar content [27]. Fruit juices, like carbonated drinks, have an acidic pH and contain sugar, making it unclear whether they require more alkaline-stimulated saliva for neutralization. Despite being perceived as a healthier choice due to their fiber and nutrient content, fruit juices can have a similar impact on dental health as carbonated drinks. Their acidity and sugar levels contribute to dental erosion, a factor often overlooked. However, despite the considerable knowledge about saliva’s composition and functions, salivary lubrication after exposure to dietary sugar and acids remains poorly understood.

This study aimed to investigate the short-term effects of consuming acidic and sugary beverages on human salivary lubrication, adsorption properties, and protein composition. Specifically, it seeks to determine how apple juice, known for its acidic and sugar composition, impacts the lubrication behaviour of saliva and the adsorbed salivary layer and whether protein composition/ concentration can explain those alterations (if any) in the tribological behaviour. Our hypothesis was consumption of sugary and acidic beverages would precipitate salivary proteins and delubricate saliva that will last for a short period. To investigate this, we assessed the temporal effects of exposure to apple juice on salivary lubrication for the first time where saliva after exposure was tested after one and 10 min. These properties are crucial in understanding saliva’s resilience and ability to revert to low friction (if at all) after being subjected to varying oral (feeding) conditions. Saliva properties such as viscoelasticity, hydration, and changes in protein composition and contents after exposure to apple juice with water as control have also been assessed. The findings of this study could offer new perspectives for developing and studying behaviours of different food and beverage constituents, artificial saliva products, and strategies to prevent tooth wear that has been reported to be caused by repeated consumption of sugary and acidic food and beverages.

## 2. Materials and methods

### 2.1. Participant recruitment

Ethical approval was obtained from the National Research Ethics Committee (North West – Preston Research Ethics Committee, REC reference: 23/NW/0075). Thirty-two healthy participants (comprising students and staff from the University of Portsmouth) were recruited between May 1, 2023, and October 19, 2023. The mean age for the participants was 37.7 years (SD = 11.7, Range 18–56). More females were recruited [27 (78.1%)] than males [5 (21.9%)]. Following informed written consent, dental and medical histories were checked as well as diet and reflux screening were performed to determine whether the participants met the inclusion criteria of this study. Participants were excluded from the study if they had dental erosion, severe dentine hypersensitivity, periodontitis or restoration of the occlusal or incisal surfaces of the upper anterior teeth and first molars, missing anterior teeth, anterior crowns/bridges or cavitated caries on more than one tooth, history of eating disorders, gastro-oesophageal reflux, xerostomia, bruxism, prescribed xerostomia/heartburn medication, pregnancy, participation in other research studies in the last 30 days, inability to speak or understand the English language and the necessity of taking antibiotics or mouthwash before dental visits.

### 2.2. Saliva collection

**Fig 1:**
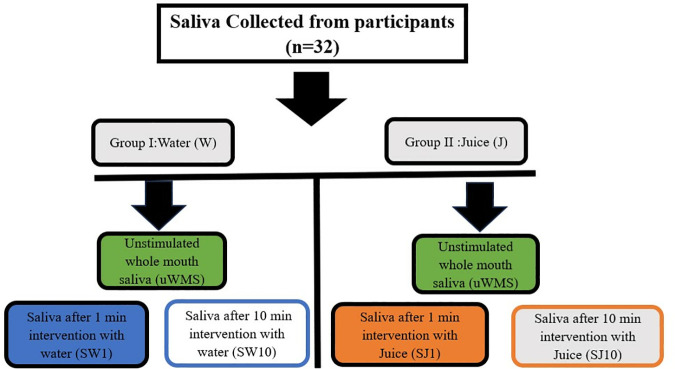
Flowchart of the saliva samples collected for the two main experimental groups: water (W) and apple juice (J) groups.

Saliva samples were collected from two groups: the control, i.e., water group (W) and the apple juice group (J), at three distinct time points [baseline (unstimulated whole mouth saliva (uWMS)), one-min (SW1, SJ1), and 10-min post-rinse (SW10, SJ10], resulting in three subgroups for each group, as illustrated in Fig 1.

Participants were instructed to abstain from eating or drinking for at least two hours before their visit. This methodology was chosen as it closely replicates the typical consumption of water and juice, ensuring a realistic representation of their effects.

#### Group I (control): Water.

On the first visit and upon arrival (9 AM −12 PM), baseline uWMS was collected from each participant via a passive drool into a pre-weighed vial and sterilized Falcon tube. Subsequently, participants were then given 10 mL of tap water (W) (Portsmouth drinking water, pH 7.5, Chloride 250 mg/L, Sodium 200 mg/L, Manganese 50 µg/l) to rinse their mouths for one min before spitting it out. Two additional saliva samples were subsequently collected: one immediately after the one-min rinse (SW1) and another 10 min post-rinse (SW10). In total, three saliva samples were collected per participant: uWMS, SW1, and SW10 as a function of time after water (control) exposure.

#### Group II: Juice.

For this group, 100% pure apple juice (pH 3.5, sugar content 111 g/L) purchased from a local supermarket (Tesco, UK), was used and was selected from among various acidic and sugary juices due to its relatively higher pH, high sugar levels and popularity as a beverage. Each participant was requested to return for a subsequent visit (visit 2), scheduled at least three days following their initial visit (Water intervention). During this session, the identical process employed in the first group was replicated, with the exception that water was substituted with apple juice (J). This resulted in the production of three saliva samples after the J exposure at three points of collection: baseline uWMS, after the one-min rinse (SJ1), and another 10 min post-rinse (SJ10).

All the saliva samples were centrifuged at 4000 × *g* for 5 min according to previous studies [28,29], immediately chilled on ice after collection, divided into 1 mL aliquots, and stored at −80 °C within 30 min. Before use, the samples were defrosted at room temperature for 3 h and pooled, this mitigates any variability between participant saliva samples from each time point, resulting in a total of six pooled samples (Fig 1).

### 2.3. Soft tribology

The frozen saliva samples (1 mL aliquot, n = 32) from six-time points (Fig 1) for both groups (W and J) were defrosted. Samples from each time point were pooled at room temperature (22 ± 1 °C), resulting in a total of six pooled samples (n = 6). Thawed saliva was mixed vigorously with a vortex mixer to resuspend the precipitation of proteins upon thawing to avoid the loss of specific proteins of less than 14 kDa, such as statherin and/or histatins [30]. The samples were run on hydrophobic tribo-pairs using a ball-on-disc set-up made with polydimethylsiloxane (PDMS) in a Mini-traction machine (MTM2, PCS instruments, UK). A normal load of 2.0 N resulted in a Hertzian contact pressure of 200 kPa and a slide-to-roll/ratio of 50% was employed [31,32]. The elastic modulus was 2.1MPa and surface roughness 20nm.

The sliding roll ratio (SRR) was calculated using equation (1)


$$SRR=\;\frac{2\;{(u}_{a,}x-\;u_{b,}x)}{u_{a,}x+\;u_{a,}x}$$

Where $u_{a,}x$ and $u_{b,}x$ are speeds of body *a* and *b* in the *x* direction. Sliding speeds starting from 3000 mm/s to 1 mm/s were employed and the temperature was maintained at 37°C ± 1 in an enclosed chamber to mimic physiological conditions. These conditions mentioned align with protocols previously reported in the literature [33,34]. A thorough cleaning protocol was implemented between each experiment to prevent surface contamination. This involved sequential 10-min sonication steps in 2 wt% sodium dodecyl sulfate (in deionised water), isopropanol, and deionised water.

The wear of the PDMS disk was carefully monitored and compared to a water baseline, which was replaced if disk became subsequently worn. Means and standard deviations were obtained from at least 3 replicate measurements (n = 3 x 1).

### 2.4. Adsorption measurements

The adsorption of saliva and the effect of juice on salivary pellicle was measured using QCM-D (E4 system, Q-Sense, Biolin Scientific, Sweden) for real-time monitoring of thin salivary pellicle and surface interactions as described in previous work [35–38]. Prefabricated gold quartz crystal with a root square mean roughness of 1nm (QSX 301, Q-Sense) was used throughout the QCM-D experiments which was cut in AT angle with a fundamental frequency of 5 MHz. Gold, being a hydrophilic surface, facilitated the adsorption of salivary proteins to hydrophilic teeth-like surfaces, which is crucial for understanding salivary film dynamics. Gold sensors were cleaned for 10 min under UV/ozone, followed by sonication in a 2% w/w sodium dodecyl sulphate solution for 15 min, rinsing and sonication in ultrapure water for 15 min, and 10 min under UV/ozone. Saliva samples to be examined were diluted and equilibrated at 25 °C before measuring. The flow rate was controlled using a peristaltic pump at a rate of 100 μL/min at 25 °C. The frequency (f) and dissipation (D) at different overtones were measured with each solution. HEPES (4-(2-hydroxyethyl)-1-piperazineethanesulfonic acid) was used as a buffer media that is widely used in biological research due to its optimal buffer capacity within the physiological range (pH 6.8–8.2). Buffers were initially injected to obtain a stable baseline reading to which saliva samples were subsequently injected until equilibrium adsorption (no change in ∆f or ∆D). Finally, the buffer was used once more as a wash to remove excess salivary proteins. Hydrated mass was calculated using 3^rd^, 5^th^, 7^th^ and 11^th^ overtones applying Voigt’s viscoelastic model [39] using “Smartfit Model” by Dfind (Q-Sense, Biolin Scientific, Sweden) software with 5th overtone represented in ∆f or ∆D figures. Three distinct protocols were implemented to examine the impact of juice on the formation of salivary pellicle using QCM-D as follows:

#### Protocol 1: Surface adsorption characteristics of apple juice (Buffer, Apple juice, Buffer).

This protocol was developed to assess the adsorption of apple juice on gold quartz crystal cells as a control. Citrate buffer was prepared by combining 10 mM citric acid monohydrate and 10 mM trisodium citrate dihydrate in appropriate proportions to match the pH of apple juice (pH 3.5). The QCM-D flow cells were first injected with citrate buffer until a stable baseline was achieved. Next, diluted apple juice (1:20 v/v ratio apple juice to citrate buffer), was then injected until fully adsorbed which was finally washed once more with the citrate buffer. Means and standard deviations for data analysis were obtained from at least 3 replicate measurements using different solutions made from start (n = 3 x 1).

#### Protocol 2: Surface adsorption characteristics of saliva and apple juice (Buffer, Saliva, Buffer, Juice, Buffer).

The second protocol mimicked the *in vivo* consumption of apple juice in which the impact of the apple juice on salivary-coated gold quartz crystal cells was assessed. In this protocol, HEPES buffer was used and prepared by dissolving 10 mM powdered HEPES in ultrapure water and adjusting the pH to match salivary pH using 1 M NaOH (pH 7.0). Firstly, the buffer solution HEPES was injected into the chamber containing gold quartz crystals until a stable baseline was achieved. Next, the baseline saliva (uWMS) diluted in HEPES buffer (2.5/100 v/v) was injected into the chamber until fully adsorbed which was then washed with HEPES to form a stable saliva-coated layer. Diluted juice (1:20 apple juice to citrate buffer), was then injected until no changes of adsorption were observed and finally rinsed with HEPES again. Means and standard deviations for data analysis were obtained from at least 3 replicate measurements (n = 3 x 1)

#### Protocol 3: *Ex-vivo* adsorption measurements.

The third protocol assessed the interaction between saliva and apple juice by pre-mixing the juice with saliva before adsorption, simulating the consumption *ex vivo*. An ex-vivo preparation was made by mixing saliva and apple juice in a 1:4 v/v ratio [30], followed by a 10-min incubation, centrifugation at 4,000x *g* for 5 min in line with previous protocols [31,34]. The pH of this solution was measured (pH 3.6) and diluted in pH 3.6 citrate buffer (1:20 v/v ratio) [34,40]. This process removed some of the proteins interacting with the juice, and the resulting adsorption behaviour of the gold crystal/salivary proteins was measured. Following a similar QCM-D protocol, citrate buffer (pH 3.6) was injected until the baseline was stable, the 1:4 saliva-apple juice solution was then introduced until equilibrium adsorption was reached, and the system was then washed with the initial citrate buffer. This procedure allowed for a detailed examination of how the components in the saliva-apple juice mixture interacted with the gold crystal surface. Means and standard deviations for data analysis were obtained from at least 3 replicate measurements (n = 3 x 1).

### 2.5. Quantification of salivary total protein concentration (TPC)

Total protein analysis was performed to quantify the protein concentration across saliva samples, providing important context for understanding the role of salivary proteins in the lubrication process. This allowed us to correlate protein content with the tribological and viscoelastic properties of the salivary film, critical for understanding its interactions with water and apple juice. Twenty randomly selected frozen saliva samples (1 mL aliquot, n = 20) from six-time points (Fig 1) for both groups (W and J) were defrosted, thawed, and vortexed as described above. The baseline uWMS from each participant (n = 20) in both groups (W) and (J) were combined to form a single group of (uWMS). This consolidation decreased the number of groups from six to five groups (uWMS, SW1, SW10, SJ1 and SJ10). The total protein content of these selected saliva samples (n = 20; 25 µl each) was determined using the bicinchoninic acid assay (BCA). Purified bovine serum albumin standard (BSA) in a concentration of (2 mg/mL) (Thermofisher Scientific, IL, USA) was used according to the manufacturer’s instructions and previously published methods [6]. In brief, the cuprous ions, produced by the reaction of protein with cupric ions under alkaline conditions, react with bicinchoninic acid forming an adduct by an addition reaction detected by measuring the absorbance of the resulting complex. A calibration curve was constructed using serial dilutions of BSA. A spectrophotometer was then used to measure the absorbance of all samples at a wavelength of 562 nm (BioRad Laboratories Ltd, Hemel Hempstead, UK). Salivary total protein concentration was calculated in µg/µL using a standard curve.

### 2.6. Sodium dodecyl sulphate polyacrylamide gel electrophoresis (SDS-PAGE)

For SDS-PAGE analysis, the total protein content of each saliva sample was equalized across the five groups within a participant’s set based on protein concentration [total of 20 participants (n = 20)]. Samples were diluted with deionized water and mixed with Laemmli sample buffer containing dithiothreitol (DTT) to reduce disulfide bonds. They were then heated to 95°C for 10 min to ensure protein denaturation, vortexed, and 10 µL of each sample was loaded into a lane of a 4–20% Mini-Protean TGX stain-free protein gel (Bio-Rad, CA, USA). Electrophoresis was performed at 140 V until complete protein separation was achieved, and gel images were captured using a Chemi Doc Gel Analyzer (Bio-Rad, CA, USA). Molecular weights of protein bands were determined using a protein molecular weight ladder (Thermo Fisher Scientific, IL, USA) and compared with previously reported salivary profiles [41,42]. Protein band intensities for six salivary proteins [mucin, cystatins, immunoglobulins (Igs) (IgG kappa chain and polymeric IgG receptor), amylase, and carbonic anhydrase (CA)] were quantified via densitometry analysis in ImageJ software [43]. To normalise values, the uWMS band intensity was set to 100% within each gel. Normalised values (%) were pooled across all gels (n ≤ 20 participants).

### 2.7. Statistical analysis

Each sample was measured in triplicate and the means and standard deviations were reported. The collected data were analysed by SPSS version 28. The normality of the distribution of the data was assessed using Shapiro-Wilk and Kolmogorov-Smirnov tests. The tribology data were normally distributed and therefore one-way ANOVA with Tukey’s adjustment was applied to determine whether significant differences existed between the means of the groups or not. Hydrated mass was calculated using the Voigt model for viscoelastic solids using “Smartfit Model” with Dfind Software (Q-sense, Biolin Scientific, Sweden) [39]. Due to the non-normal distribution of protein expression data for SDS-PAGE, we conducted non-parametric statistical analyses on the data among the experimental groups. Mann-Whitney U test was used to compare protein expression between two independent groups. Post-hoc Bonferroni correction analysis was conducted to identify specific group pairs exhibiting significant differences in protein expression.

## 3. Results and discussion

### 3.1. Tribology

**Fig 2:**
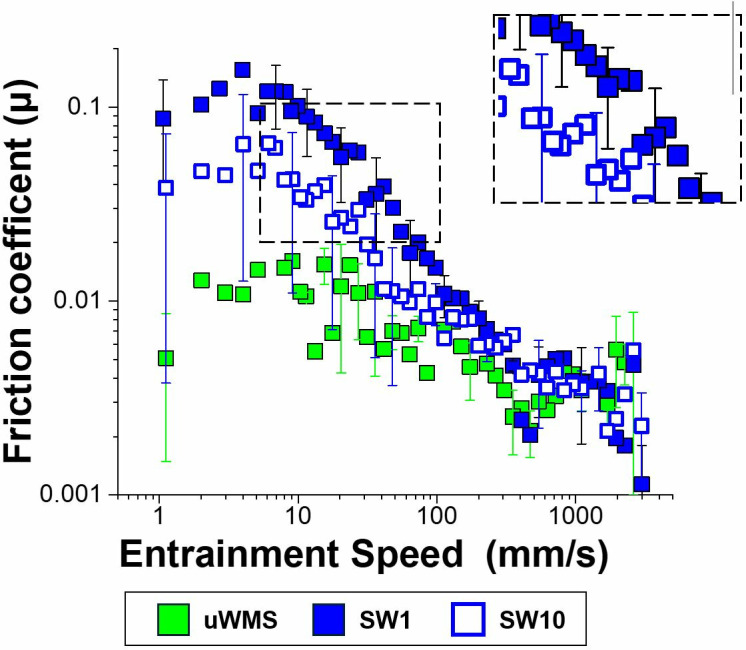
Mean friction coefficient (µ) of hydrophobic surfaces in the presence of unstimulated whole-mouth saliva (uWMS), saliva after intervention with water [one-min intervention (SW1), and 10-min intervention (SW10)] as a function of entrainment speed. Error bars represent standard deviations of three readings on pooled saliva (n = 32).

**Saliva lubrication**. Fig 2 shows the friction coefficient (µ) of hydrophobic pairs of polydimethylsiloxane siloxane (PDMS) of tribometer as a function of entrainment speed of uWMS and saliva after one-min (SW1) and 10 min (SW10) intervention with water.

The baseline uWMS from each participant (n = 32) in both groups (W) and (J) were combined to form a single group of (uWMS). A boundary lubrication regime with a very low friction coefficient (µ ~ 0.01) was observed for uWMS at speed ≤ 40 mm/s. Particularly at speeds > 100 mm/s, a transition towards a mixed lubrication regime was apparent with an order of magnitude decrease in µ to ~0.002. At speeds of 625 mm/s, a further onset of hydrodynamic lubrication was observed. The observed friction trend of uWMS, along with the low magnitude of friction coefficients (µ), aligns with findings previously reported in the existing literature [8,14,44]. Friction coefficients for saliva and saliva mimics documented in previous studies range between μ ≈ 0.02 and 0.45. This indicates that our results for uWMS fall within the lower end of this reported range [9,15,44].

**Fig 3:**
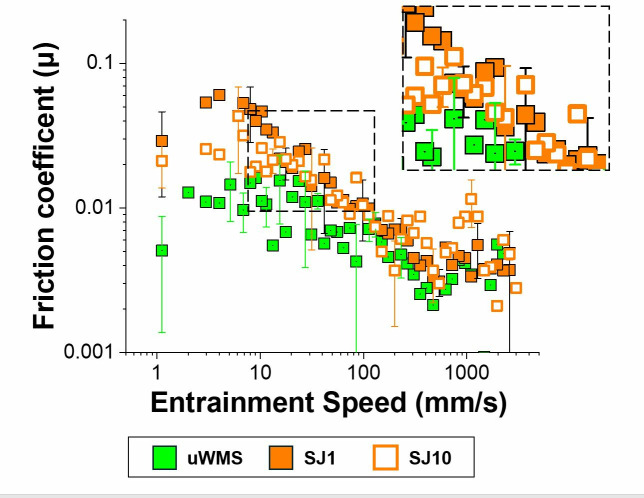
Mean friction coefficient (µ) of hydrophobic surfaces in the presence of unstimulated whole-mouth saliva (uWMS), saliva after a one-min intervention with apple juice (SJ1), and saliva after a 10-min intervention with juice (SJ10), as a function of entrainment speed. Error bars represent standard deviations of three readings on pooled saliva (n = 32 subjects).

**Comparison of apple juice versus water (control) on delubrication of saliva**. We then turned our attention to examining the frictional behaviour of uWMS versus saliva samples following water intervention (Fig 2) and compare with the juice intervention [after one min (SJ1) and 10-min (SJ1) (Fig 3).

Strikingly, irrespective of water or juice intervention, the friction coefficients were higher in the boundary and mixed regimes with overlapping hydrodynamic lubrication regimes. Particularly, in case of water intervention, the friction increased by an order of magnitude which might not be intuitive and then started to reduce when the salivary friction was measured after 10-min (Fig 2). Strikingly, in case of juice intervention, the friction coefficients although higher than uWMS were lower than water intervention (Fig 3). Irrespective of water or juice consumption, friction measured after 10-min showed that it tended to revert to uWMS particularly in the boundary regimes (1–10 mm/s) highlighting the rather transient nature of delubrication.

**Fig 4:**
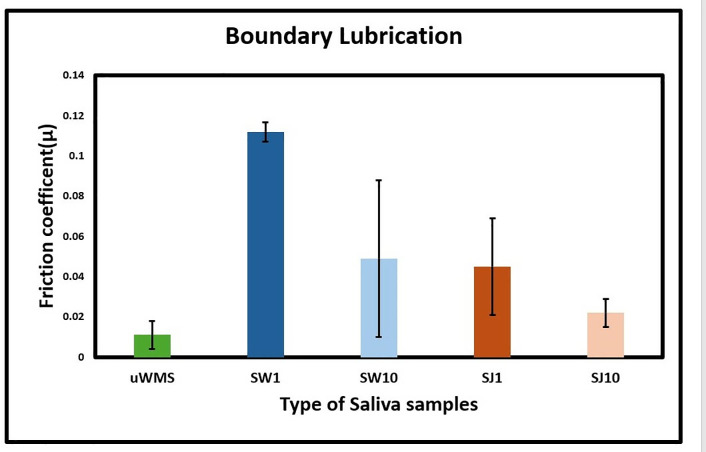
Mean friction coefficient of hydrophobic surfaces (boundary regime) in the presence of unstimulated whole mouth saliva (uWMS), saliva after a one-min intervention with water (SW1), saliva after a 10-min intervention with water (SW10), saliva after a one-min intervention with juice (SJ1) and saliva after a 10 -min intervention with juice (SJ10), as a function of entrainment speed. Error bars represent standard deviations of three readings on pooled saliva (n = 32 subjects). Figs 2–4 and Table 1 show that the friction coefficient (μ) of uWMS (0.011) was significantly lower (*p < 0.05*) than that of saliva after one min of intervention with either water (SW1: 0.112) or apple juice (SJ1: 0.045). uWMS, primarily derived from the submandibular and sublingual glands, is rich in mucins—heavily glycosylated proteins that retain water and form a hydrated biogel. This biogel containing mucinous proteins as well as cationic proteins contributes to boundary lubrication by preventing direct surface contact [5,18,19,44,45]. The observed boundary lubrication with uWMS in this study likely involved a salivary pellicle adsorbed onto one or both sliding surfaces, effectively reducing the coefficient of friction (µ).

To understand the statistical differences better, Table 1 shows the means of friction coefficients at varying speeds of the five saliva groups (uWMS, SW1, SW10, SJ1 and SJ10). Fig 4 illustrates the boundary lubrication behaviour across the five saliva groups.

**Table 1:** Means and standard deviation (SD) of the friction coefficient (µ) of the five saliva samples in each boundary, mixed, and hydrodynamic regimes.

Saliva samples	Boundary lubrication (10 mm/s)	Mixed lubrication (50 mm/s)	Hydrodynamic lubrication (1000 mm/s)
**uWMS**	**0.011** ^**ab**^	**0.006** ^**ab**^	**0.003**
**SW1**	**0.112** ^**abdc**^	**0.026** ^**abcd**^	**0.003**
**SW10**	**0.049** ^**a**^	**0.013** ^**c**^	**0.003**
**SJ1**	**0.045** ^**bd**^	**0.012** ^**b**^	**0.004**
**SJ10**	**0.022** ^**c**^	**0.011** ^**d**^	**0.003**

Means and standard deviation (SD) of the friction coefficient (µ) of the five saliva samples in each boundary, mixed, and hydrodynamic regimes. The superscripts, a, b, c, d, and e, denote specific pairwise comparisons between groups or conditions. Each superscript represents a different comparison, indicating whether there was a statistically significant difference between the corresponding pairs of data. The same superscript in the same column indicates a statistically significant difference (p < 0.05). For example, SW1 (0.112ᵃᵇᵈᶜ) is significantly different from all other groups, as it shares at least one superscript with each—“a” with SW10, “b” with uWMS and SJ1, “d” with SJ1, and “c” with SJ10—indicating statistically significant differences (p < 0.05) within the same column.

Strikingly, a significant increase (*p* *<* *0.05*) in the friction coefficient (µ) to 0.1 was observed following a one-min water rinse (SW1), indicating a strong delubrication effect purely by water (Fig 2). The boundary lubrication in water was notably disrupted, with mixed lubrication occurring at a faster speed (50 mm/s). Interestingly, despite a reduction in friction (µ) values in the boundary lubrication in SW10, it remained higher (0.049) than uWMS. This suggests that while water-induced delubrication is transient, it does not fully restore the lubricating properties of uWMS after 10 min, likely due to protein dilution or partial desorption of the pellicle. Nevertheless, the decrease of friction by over 50% (from 0.112 to 0.049) suggests that water delubrication effect is tending towards the uWMS as a function of time.

Shifting our focus to juice (J) consumption, saliva exposed to apple juice after one min (SJ1) showed a four-fold increase in boundary friction (µ = 0.045) compared to uWMS (*p* *<* *0.05*), but it was still significantly lower than that of water (SW1) (*p* *<* *0.05*). After 10 min of juice intervention (SJ10), there was a 50% reduction in friction (µ = 0.022) and almost reverted to uWMS (*p* *>* *0.05*), indicating that J incorporation had a transient delubrication effect. This suggest that although we partially validate our hypothesis that juice might have a delubrication effect upon short-term consumption but such effect is fairly transient. Despite having a neutral pH (7.5), the tap water used in this study, with its ionic content, may have caused a stronger delubrication effect by disrupting the hydration of salivary proteins, particularly mucins. Mucins depend on their highly hydrated glycoprotein structure to form a lubricating, viscoelastic film. Increased ionic concentration can destabilize this hydration, leading to mucin aggregation or collapse, which compromises film integrity and increases friction. This concept aligns with observations by [46], who found that raising ionic concentration affected the lubrication performance of mucin films through altered hydration and ion-binding dynamics. Also, minerals may have stimulated the parotid salivary glands to produce slightly more watery saliva to balance the ionic load, further enhancing delubrication [47]. Although we included uWMS as a baseline control, which helped account for natural variability in salivary composition, and also, SDS-PAGE analysis showed no significant changes in mucin and amylase levels across all groups, suggesting protein quantity was maintained, it remains possible that the increased friction observed after the water rinse was partially due to transient dilution effects. Tribological measurements were conducted immediately after the intervention, during which time the concentration of lubricating macromolecules such as mucins may have been temporarily reduced. While total protein concentrations were equalized for SDS-PAGE analysis, the rheological and tribological properties of saliva depend not only on protein quantity but also on the concentration and conformation of specific glycoproteins. Future studies should consider incorporating controlled dilution protocols to better separate the effects of physical dilution from true compositional changes.

In contrast, apple juice, with limited ionic content, had a less effect unlike water. Its acidic pH and sugary components likely contributed to some degree of alterations in protein composition and adsorption within the salivary pellicle, reducing lubrication (discussed later), however the effect is less pronounced as compared to water. The findings in this study are consistent with previous studies, especially when saliva is exposed to acidic conditions [15,18], underscoring the sensitivity of salivary lubrication to even minor alterations in pH, which can affect the protein structure and function of the salivary pellicle over time. Previous tribology studies have also shown that pH significantly correlates with the rate of friction increase in saliva, even with weak acids such as lactic acid, malic acid, and tartaric acid [48]. Exercise-induced changes in salivary flow and composition, such as increased protein secretion (e.g., mucins), have also been found to enhance lubrication, highlighting the sensitivity of salivary lubrication to compositional alterations [28]. However, what we find uniquely is that such delubrication effect by a sweetened sugary beverage only lasts for few min and revert to salivary lubrication. Another event observed was that there were no significant differences in the coefficient of friction (µ) values observed between SJ1, SJ10, and uWMS in mixed and hydrodynamic lubrication, aligning with studies showing salivary proteins reduce friction in boundary and mixed lubrication, with µ dropping to ~0.002 as speed increases [9,19,31,49,50]. To understand better the behaviour upon J consumption, the film properties were probed real-time using QCM-D.

#### Quartz Crystal microbalance with dissipation (QCM-D).

**Fig 5:**
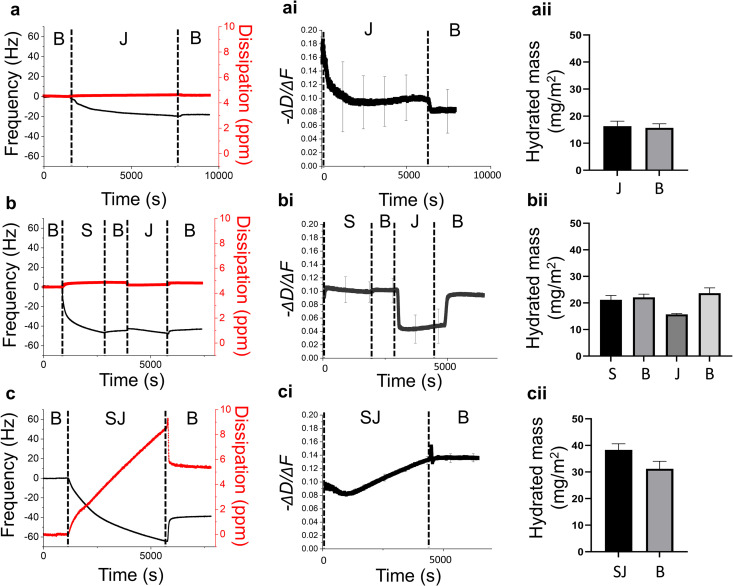
Frequency and dissipation shifts (5^th^ overtone) as a function of time and calculated hydrated mass changes in response to buffer (B), saliva (S), apple juice (J), and saliva-juice mixture (SJ) on hydrophilic gold surfaces. Adsorption characteristics in apple juice (a) refer to protocol 1 in methods, influence of apple juice on saliva (b) refer to protocol 2 in methods or *ex-vivo* adsorption measurements (c) refer to protocol 3 in methods. Corresponding viscoelastic changes (-∆D/∆f) are presented in ai, bi and ci and hydrated mass (mg/m²) calculated using 3^rd^, 5^th^, 7^th^ and 11^th^ overtones are presented in aii, bii and cii for the respective protocol. Means with error bars representing standard deviations of at least three readings (n = 3 x 1).

Fig 5 shows the changes in salivary film properties of uWMS with and without apple juice in the three protocols as described in section 2.4. The changes in frequency (∆f) and dissipation (∆D) were measured providing quantitative information on the adsorption kinetics, mass, and viscoelastic properties of the adsorbed salivary film [39]. The changes in frequency (∆f) indicate mass changes, where frequency is inversely proportional to adsorbed mass. Dissipation shifts (∆D) reveal viscoelastic changes, with higher ∆D indicating more elastic films and lower ∆D indicating more ridge films. The parameter -∆D/∆f was also used to indicate the film’s viscoelastic properties. A lower value indicates a rigidly adsorbed layer with minimal dissipation relative to frequency shift (less viscoelastic layer), while a larger value corresponds to a soft, viscoelastic layer with high dissipation relative to frequency shift.

In the first protocol (Figs 5a, 5ai and 5aii), three solutions—citrate buffer (B), diluted apple juice (J), and a final buffer rinse (B)—were sequentially introduced over the crystals. The introduction of apple juice resulted in a frequency shift from 0 Hz to −20 Hz (Fig 5a), which remained even after rinsing, indicating mass adsorption. This is likely due to the small amounts of proteins and polyphenols naturally present in the apple juice concentrate. This layer was also rigid as observed with minimal -∆D/∆f shifts (Fig 5ai), forming a compact non-elastic film. The average adsorbed mass of apple juice was 16.33 ± 1.8 mg/m², which showed no significant change after the final rinsing with citrate buffer (15.7 ± 1.5 mg/m²). This confirms that the layer formed was stable and not easily removed, but also indicates minimal water content or structural rearrangement—consistent with the formation of a sparse, rigid, and relatively non-swollen film. In the second protocol (Figs 5b,5bi and 5bii), a baseline salivary film was first adsorbed onto the sensor, confirmed by a ∆f decrease (~−40 Hz) and a ∆D increase (~5 ppm) with high degree of hydrated mass (21.2 ± 1.6 mg/m²). Subsequent rinsing with HEPES buffer caused only minor changes, indicating that the salivary film was strongly adsorbed (Fig 5a and 5ai). Upon exposure to apple juice, ∆f showed only slight decrease, while ∆D significantly increased (*p* *<* *0.05*, Fig 5a) reflecting in a loss of viscoelasticity in -∆D/∆f (Fig 5ai). This suggests that while little additional material from the juice was adsorbed, the existing viscoelastic, hydrated salivary layer transitioned into a more compact and rigid structure. Hydrated mass data (Fig 5bii) further supports this transition. The film initially had a mass of 21.2 ± 1.6 mg/m², which slightly increased to 22.1 ± 1.2 mg/m² after rinsing. During juice exposure, the mass temporarily decreased to 15.7 ± 0.3 mg/m², likely due to structural compaction or component displacement, followed by swelling to 23.7 ± 2.0 mg/m² after the final rinse, indicating a reversible restoration of viscoelasiticty, demonstrating the resilience of saliva under varying pH conditions. This finding fully aligns with our tribology results (Fig 3, Table 1), where apple juice might aggregate salivary proteins leading to higher adsorbed mass and higher friction but such aggregation may reduce post rinsing with buffer (Fig 5b i). The juice used in this study had an acidic pH of 3.5, mainly due to organic acids such as malic and citric acid. While the exact concentration of polyphenols was not provided by the manufacturer, typical values for commercial apple juices range between 50–100 mg/L. These components may have influenced the adsorption kinetics and viscoelastic properties of salivary films with sugars promote salivary film formation through hydrogen bonding, organic acids affect protein structure and charge by lowering pH, and polyphenols bind and cross-link proteins, together enhancing the stability and viscoelastic properties of the saliva–juice film.

For the final protocol (Figs 5c and 5ci), we passed a centrifuged supernatant of apple juice mixed with saliva (i.e., an *ex vivo* boli) through the system (SJ), investigating the role of soluble, non-interacted proteins in the adsorption process and how they contribute to the final structure of the salivary pellicle. We aimed to isolate the effects of free proteins that had not previously interacted or precipitated. Interestingly, we observe a continuous linear decrease in frequency (∆f) and increase in dissipation, suggesting ongoing protein adsorption, which may be driven by their zwitterionic nature and ability to reorient on the surface, facilitating sustained binding. This final QCM-D protocol helped reveal the role of small molecular proteins in sustained adsorption rather than mucins alone. Hydrated mass data (Fig 5cii) support this sustained adsorption process. The film reached a mass of 38.3 ± 2.3 mg/m² during the exposure phase to saliva-juice mixture, which decreased only moderately to 31.2 ± 2.8 mg/m² after buffer rinsing. This pattern indicates that a substantial portion of the salivary-juice mixture remained irreversibly adsorbed, forming a soft, hydrated, and dynamically structured layer. The result highlights the unique role of smaller soluble proteins and juice components in enhancing film mass and viscoelasticity beyond what is typically observed with mucin-rich native saliva alone. Since mucins are large, heavily glycosylated proteins, they may have interacted with apple juice components and been centrifuged out, leaving behind smaller soluble proteins in the supernatant. This was confirmed by the SDS-PAGE analysis later, which showed that mucin expression levels remained unchanged between the water and juice groups, while smaller salivary proteins exhibited differences, which might not have had large sustained effects on delubrication.

This behaviour has been previously reported [51,52] and may indicate the adsorption of small proteins in the supernatant, which continuously reorients to facilitate sustained adsorption. Notably, upon rinsing with HEPES buffer, there is a brief, rapid increase in frequency (∆f) and decrease in dissipation (△D), suggesting that in the absence of flow, residual proteins quickly bind and interact with the existing layer which forms a final resilient viscoelastic layer. The formation of this lubricating layer is essential for oral activities [53] and is most likely influenced by small molecular proteins and their interaction with external substances rather than just mucin [48]. Previous studies have also shown that carbonated water disrupts the salivary pellicle more effectively than non-carbonated water, leading to increased friction and compromised lubrication properties [51]. Therefore, these findings also emphasize the significant role of surface properties—whether hydrophilic or hydrophobic—on how saliva interacts with food and beverages in the oral environment. Hydrophobic surfaces, like PDMS, have led to greater disruption of the salivary pellicle when saliva was rinsed with ionised water, increasing friction, while apple juice has enhanced pellicle adsorption and stability, improving lubrication. Furthermore, while the reversible changes in the salivary film observed after the HEPES rinse suggest that rinsing with deionised water following apple juice consumption may help rebalance pH and promote the reformation of the viscoelastic salivary film, it is important to recognize that the oral environment is complex. Therefore, these findings should be interpreted cautiously, and further in vivo studies are needed to fully understand the effects of rinsing on salivary film dynamics and oral health, particularly in relation to mitigating the erosive effects of acidic and sugary beverages.

### 3.2. Total protein concentration (TPC)

**Fig 6:**
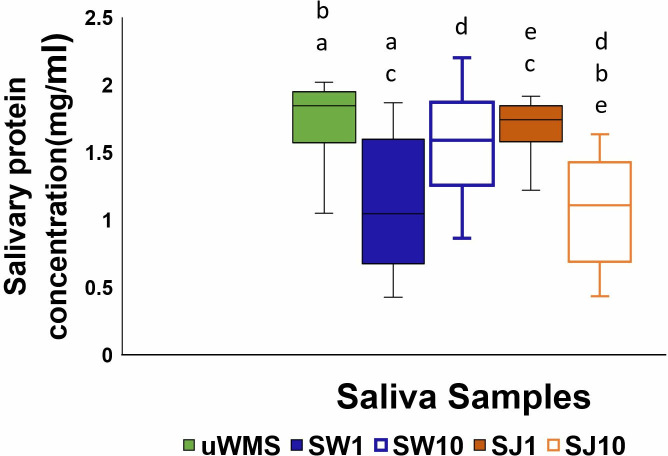
Mean and interquartile range (IQR) of total protein concentration (TPC) using BCA analysis across five saliva groups (n = 20). Identical letters denote significant differences (p < 0.05).

Fig 6 shows the mean and interquartile range (IQR) of the total protein concentration (TPC) of the five saliva groups (n = 20). Overall, the greatest mean TPC (IQR) observed was for the uWMS group (1.67 mg/mL) while the lowest mean TPC (IQR) was for saliva after 10 min of intervention with apple juice (SJ 10) (1.084 mg/mL).

Significant differences (*p* *<* *0.05*) in TPC were observed across the various groups. uWMS had a significantly higher TPC [1.67 (0.38) mg/mL, (p ≤ 0.05)] compared to SW1 [1.11 (0.92) mg/mL] and SJ10 [1.08 (0.73) mg/mL], both showing significant reduction in TPC (*p* *<* *0.001*). uWMS also showed higher TPC than SW10 [1.55 (0.62) mg/mL] and SJ1 [1.63 (0.27) mg/mL] but this was not statistically significant (p ≥ 0.05). This result suggests that the uWMS sample had a higher protein content, which correlates with better lubrication properties, as indicated by the tribology results where uWMS showed lower frictional properties compared to all other samples (Table 1, Figs 2-3). This finding also aligns with a previous study, which reported that stimulation with food-grade citric acid resulted in a significantly lower TPC compared to uWMS [54].

When comparing water and J interventions, the TPC for SW1 was significantly lower than that of SJ1 (*p* *<* *0.05*), which was consistent with the tribology results showing that delubrication by water µ = (0.112) exhibited significantly higher friction properties than SJ1 (0.045) (*p* *<* *0.05*). Additionally, the TPC of SW10 was significantly greater than that of SJ10 (*p* *<* *0.05*), but in tribology, SJ10 demonstrated better lubrication, though this difference was not significant (*p* *>* *0.05*, Table 1).

Regarding the duration of the intervention (one or 10 min), the one-min water intervention (SW1) had significantly lower TPC than SW10, whereas SJ1 had significantly higher TPC than SJ10 (*p* *<* *0.05*). This is in line with tribology data, as indicated in Table 1, where SW1 and SJ1 showed higher friction coefficient (µ) than that of SW10 and SJ10 respectively (*p* *>* *0.05*, Table 1). Apple juice, with its acid and sugar contents, may have altered protein solubility, leading to a lower TPC in SJ10 over time. Small salivary proteins like statherin, cystatins, and histatins have been found to contribute to the lubrication properties observed during the juice intervention, supporting mucin’s lubricating function [4,5,55]. These proteins are more stable and effective in acidic conditions, allowing them to maintain lubrication even with lower TPC. This could explain why SJ10 regained better lubrication similar to uWMS (Fig 3), as indicated by the tribology results, despite its lower TPC. This suggests that lubrication is not solely dependent on TPC but may be more influenced by specific proteins, especially those that are stable in acidic environments.

### 3.3. Sodium dodecyl sulphate polyacrylamide gel electrophoresis (SDS-PAGE)

**Fig 7:**
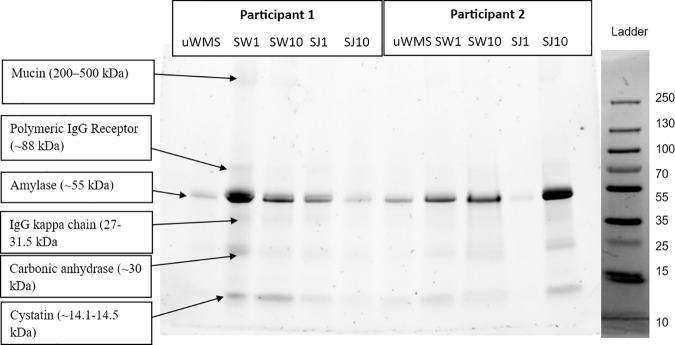
Exemplar SDS-PAGE profile shows the protein expression of six salivary proteins across five saliva groups (uWMS, SW1, SW10, SJ1, and SJ10) from two participants. A molecular weight ladder is displayed on the right-hand side for reference.

**Fig 8:**
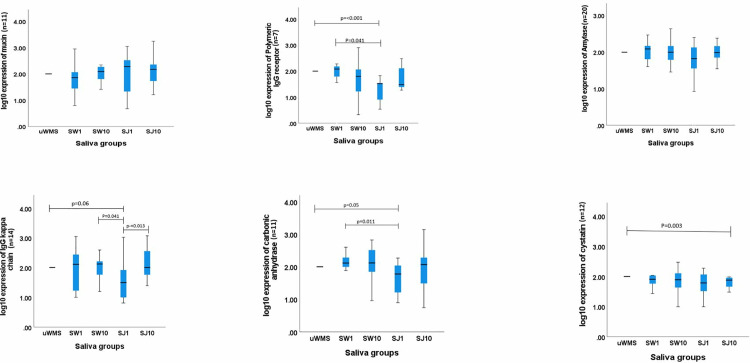
Log10 protein expression levels of the six salivary proteins across five saliva groups (uWMS, SW1, SW10, SJ1, and SJ10), as determined by SDS-PAGE analysis. Significant differences and corresponding p-values are provided.

Figs 7 and 8 show protein expression in the five saliva groups. The SDS-PAGE analysis revealed distinct protein expression patterns across saliva groups, particularly in J-treated samples. These results align with the observations from the tribology and TPC findings, shedding light on how apple juice intake impacts salivary protein expression and lubrication.

A key finding was that although mucin has been expressed in all five groups, there was no significant difference in its expression (*p* *>* *0.05*), which agrees with previous studies that while mucin is a key lubricating macromolecule, other small salivary proteins also play an essential role in achieving the low friction observed in human saliva and the salivary pellicle [55,56]. Mucins contribute to the formation of a hydrated, lubricating film that reduces friction and protects oral surfaces [48].

Furthermore, cystatin levels showed a significant reduction (*p* *<* *0.05*) after 10 min of J consumption. The decrease in cystatin levels after this short-term exposure to J is thought to reflect a physiological response to acidic conditions, as cystatins, such as cystatin B and S, have a high affinity for dental enamel and can become incorporated into the salivary pellicle during acid attack [57,58]. This depletion suggests that cystatins might play a role in enamel protection and may be involved in preventing dental erosion. This agrees with previous findings that cystatins, especially in acidic environments, bind to enamel, with pH-induced conformational changes enhancing their potential for use in dental care products [59]. Future studies should separately examine the roles and expression of Cystatin B and Cystatin S to explore their implications in dental health, as their varied expression patterns may provide valuable insights.

Amylase, on the other hand, has been consistently expressed across all groups but did not show significant differences in expression (*p* *>* *0.05*) following water and J consumption. While amylase is known for its role in lubrication by breaking down starches, the absence of major changes in its expression suggests that other factors, such as the pH of the juice does not affect amylase in short term [60,61]. Another notable finding from the SDS-PAGE analysis was the significant decrease (*p* *<* *0.05*) in CA and immunoglobulin (Ig) levels (IgG kappa and polymeric IgG) immediately after J intake. This suggests that CA may have been mobilised as part of the immediate salivary buffering response to counteract the acidic nature of the juice. The subsequent return of CA to normal levels after 10 min may indicate a transient adjustment, where the salivary system stabilises the oral pH. The reduction in Ig levels may have clinical significance, as Igs, particularly those present in saliva, play a vital role in oral immune defence and may be linked to the activation of taste receptors, which influence salivary protein secretion [62]. This modulation of immune function by gustatory stimulation, as seen in response to apple juice, underscores the complex interaction between diet and oral immune responses. The decrease in Ig levels in saliva could indicate that dietary components, like those found in apple juice, may shape the immune defence mechanisms in the oral cavity, possibly altering the overall immune response [62], or could also be related to changes in salivary secretion patterns or other factors, such as the effects of sugar or acidity [63–65] which warrant further investigation.

In summary, our SDS-PAGE analysis showed significant decreases in proteins such as cystatins, CA, and Igs following J intervention. These proteins play essential roles in the structural and functional integrity of the salivary film. The proteins in the salivary pellicle may be selectively affected by juice consumption, influencing the structure and function of the salivary pellicle. It is well documented that Statherin facilitates the initial adsorption of proteins onto enamel, forming a stable pellicle layer that helps regulate mineral homeostasis [4,6,49,59]. However, why such reduction does not directly affect the tribological properties warrants further investigation into the mechanisms contributing to these changes by comparing the water used in this study (Portsmouth hard water, known for its high ion content) with deionized water. Some studies [6,66] found a significant reduction in TPC in the salivary pellicle of patients suffering from dental erosion, while other research, including one by Zhang et al., [67] found no significant difference in TPC between unstimulated and stimulated saliva.

Building upon this, further proteomic analyses, including mass spectrometry and targeted western blots for Cystatin B and Cystatin S, are underway by our research group. These studies aim to provide a more detailed understanding of how specific salivary proteins respond to juice consumption and contribute to lubrication, pellicle formation, and enamel protection. To better elucidate these mechanisms, future studies should also quantify protein levels both in whole saliva and in the salivary pellicle before and after acidic challenges, alongside assessing the binding forces of these proteins to enamel surfaces using techniques such as atomic force microscopy (AFM).

A limitation of the TPC analysis in this study was the small sample size (n = 20), and the use of a homogeneous population of healthy individuals, which may limit the generalisability of the results, as individual variability in salivary composition and responses to dietary interventions could affect the outcomes. While our results provide valuable initial insights into the interactions between apple juice and the salivary film, future research with larger participant cohorts and more diverse populations is needed to validate and further explore these findings. Having said that, our findings highlight that short-term dietary interventions can modulate salivary proteins such as cystatins and carbonic anhydrase, both important for enamel protection and lubrication. These results have potential implications for caries and erosion prevention and oral health monitoring. Previous research has shown that salivary composition differs between caries-free and caries-experienced individuals, with compounds like citrate and ethanolamine emerging as potential biomarkers [68]. A combined biomarker approach appears more effective for risk stratification. Future studies should adopt longitudinal designs that integrate host, microbial, and behavioural factors, and utilize advanced techniques such as NMR spectroscopy, atomic force microscopy (AFM), and surface-enhanced Raman spectroscopy (SERS) to explore the molecular interactions between salivary film components and dietary acids. This approach will enhance understanding of salivary biomarkers and their roles in caries progression and enamel protection. Our findings further underscore the protective role of the salivary film in oral health and that disruption of the salivary pellicle by acidic beverages like apple juice may impair its buffering, lubricating, and barrier functions, thereby increasing susceptibility to erosion and caries. Notably, the partial recovery of the film following rinsing highlights a potential mechanism for mitigating such effects. These insights suggest that dynamic changes in the salivary film could be linked to caries and erosion risk, offering novel avenues for both diagnostic and preventive strategies. Specifically, enhancing or supplementing protective salivary proteins such as cystatins and carbonic anhydrase, both of which were affected by juice consumption, could support enamel protection and buffering. These proteins could serve as targets for functional oral care formulations, such as protein-enriched rinses or lozenges designed to replenish or stimulate protective salivary components. Also, they may act as biomarkers for early detection of dental erosion and caries risk, supporting more personalised and preventive oral health strategies. Public health campaigns and dental professionals could also recommend rinsing with water or neutral solutions after acidic drink consumption to reduce enamel erosion.

Also, it is important to emphasise that SDS-PAGE shows protein expression levels but not enzymatic activity. Amylase activity, important for carbohydrate digestion, for instance, and may also contribute to lubrication, was not assessed here. Future studies should explore how apple juice affects salivary protein functions to better understand its impact on saliva lubrication.

Another potential limitation of our ex vivo preparation is the possible removal of loosely bound salivary proteins due to sample processing steps. No direct protein assays were performed to quantify this effect. Future studies should incorporate quantitative protein profiling to more accurately assess how ex vivo procedures impact salivary protein composition. Also, different sweetened acidic beverages vary in pH, sugar content, acid types, and additives, which could lead to differing effects on salivary proteins and lubrication, which can affect salivary protein responses differently; thus, while our findings on apple juice are valuable, they may not fully apply to other drinks.

## 4. Conclusion

In this study, we aimed to understand how consumption of apple juice, a known sweetened acidic beverage affects salivary lubrication. Our findings show that apple juice consumption although affects lubrication performance immediately but has a limited effect on human saliva’s lubrication properties and viscoelasticity due to its transient effect. To our knowledge, this is the first report to specifically investigate the phenomenon of salivary delubrication following short exposure to apple juice, using a combined biophysical and biochemical approach. By integrating tribological analysis, QCM, TPC, and SDS-PAGE, we provide new insights into how sweetened acidic beverages transiently alter the lubrication properties and viscoelasticity of saliva. Apple juice formed a tightly adsorbed, rigid layer on the gold crystals, whereas saliva produced a thick, soft, and highly viscoelastic pellicle. However, the salivary layer reverted to its original viscoelastic state upon buffer reintroduction, with minimal loss of hydrated mass, highlighting the resilience of saliva when exposed to low-pH sugary juice. This fits with the TPC and SDS-PAGE results which revealed changes in protein concentration and composition. Unlike water, a one-min intervention with apple juice showed no change in TPC but resulted in a significant decrease after 10 min. This was reflected by individual proteins where changes in cystatin, CA and immunoglobulin expression were observed reflecting both the buffering and protective roles of saliva, as well as the influence of dietary components on lubrication and immune function. These findings underscore the dynamic nature of human saliva composition and its role in lubrication, buffering acids and protecting tooth enamel, especially regarding the timing of dietary sugar and acid exposure. However, this study has some limitations, including the use of pooled saliva samples, the use of ionised water as control, short intervention durations, and in-vitro conditions using non-representative materials [13]. Additionally, measuring the viscosity and viscoelasticity of saliva would have provided valuable complementary insights. Although the system became dilute following juice addition, characterising viscosity remains an important consideration for future research. To address current limitations, future studies should incorporate factors such as individual variability in saliva, the use of deionised water, extended intervention periods, hydroxyapatite sensors, and in vivo-formed pellicles to more accurately replicate natural oral conditions. Overall, the study suggests that the consumption of beverages such as apple juice can affect oral health transiently, warranting further research to explore the long-term repeat exposure effects in detail, and their implications for maintaining oral health.

## Supporting information

S1 File blots-gel images 15-7-25 pdf final. (PDF)
