# Supplementary material for: Short-term effects of sweetened acidic beverages consumption on human saliva: Colloidal properties and protein composition
Source: PLoS One. 2025 Sep 3;20(9):e0330023. doi: 10.1371/journal.pone.0330023 (PMC12407418; doi:10.1371/journal.pone.0330023)
Supplement: S1 File — (PDF) [file pone.0330023.s001.pdf]

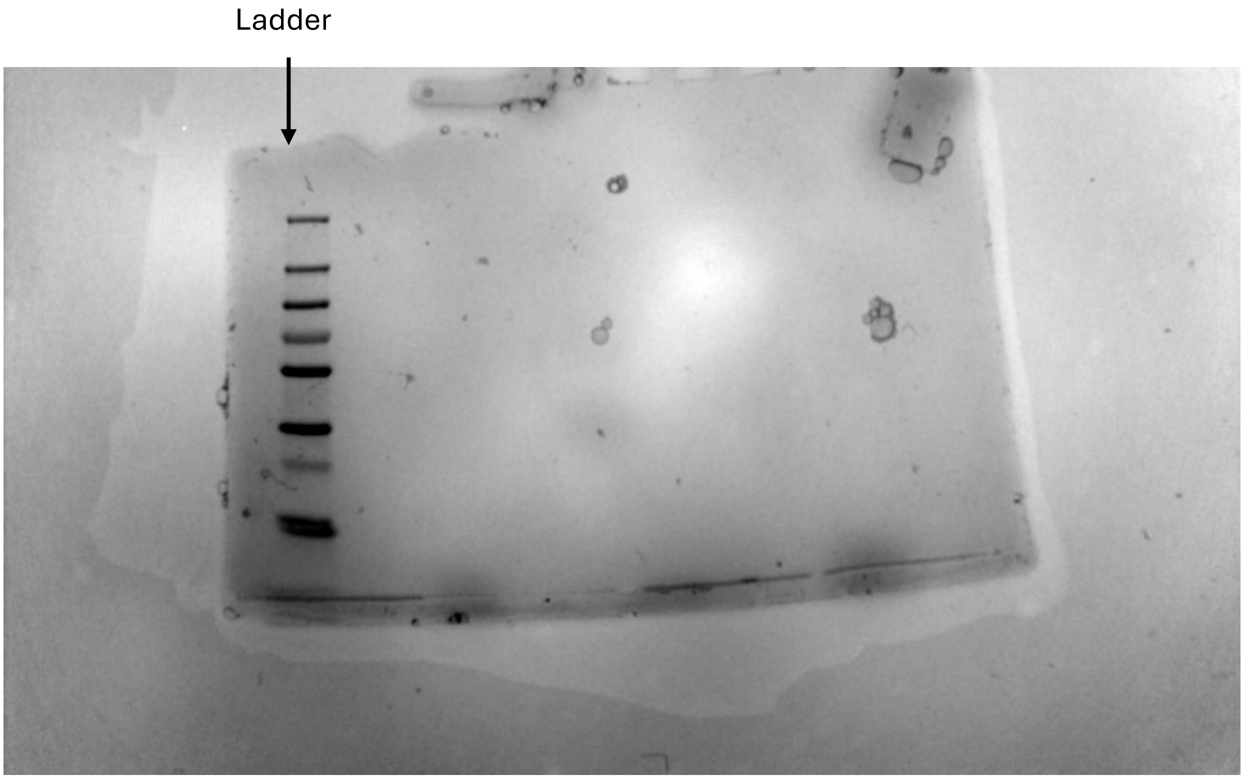

Figure 1:Ladder used to compare molecular weight of bands expressed in gels.

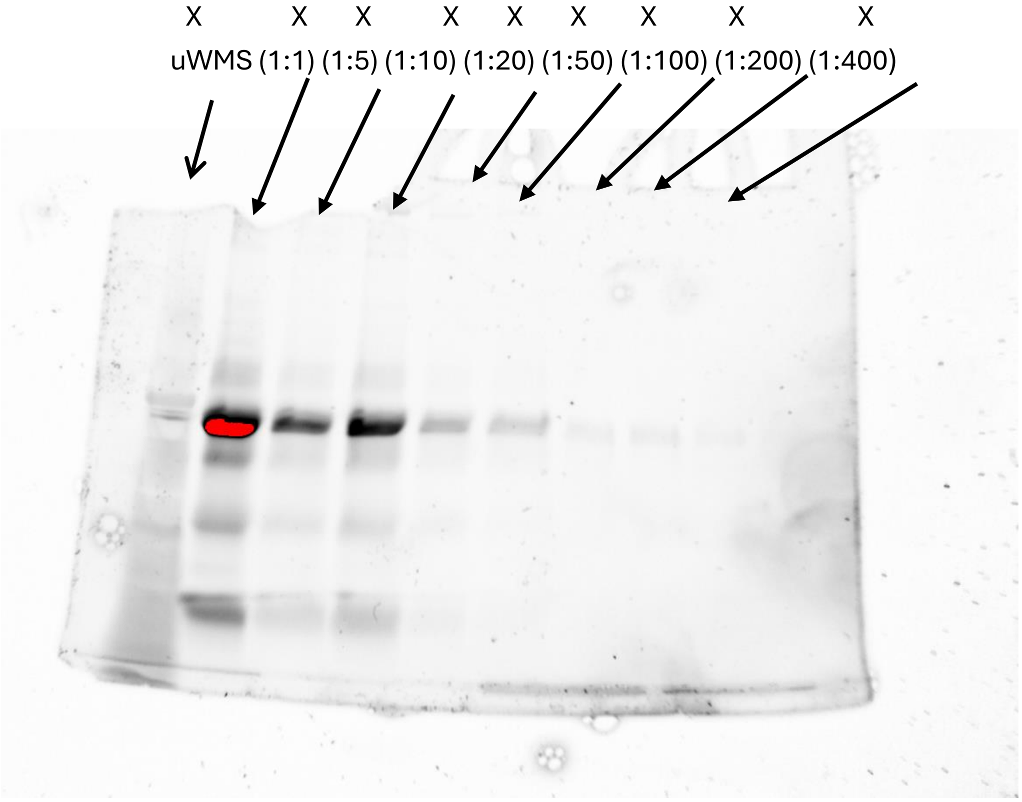

Figure 2: Experimental gel to see expression of uWMS by dilution with deionized water by which we established 1:5 dilution is appropriate to visualize bands.

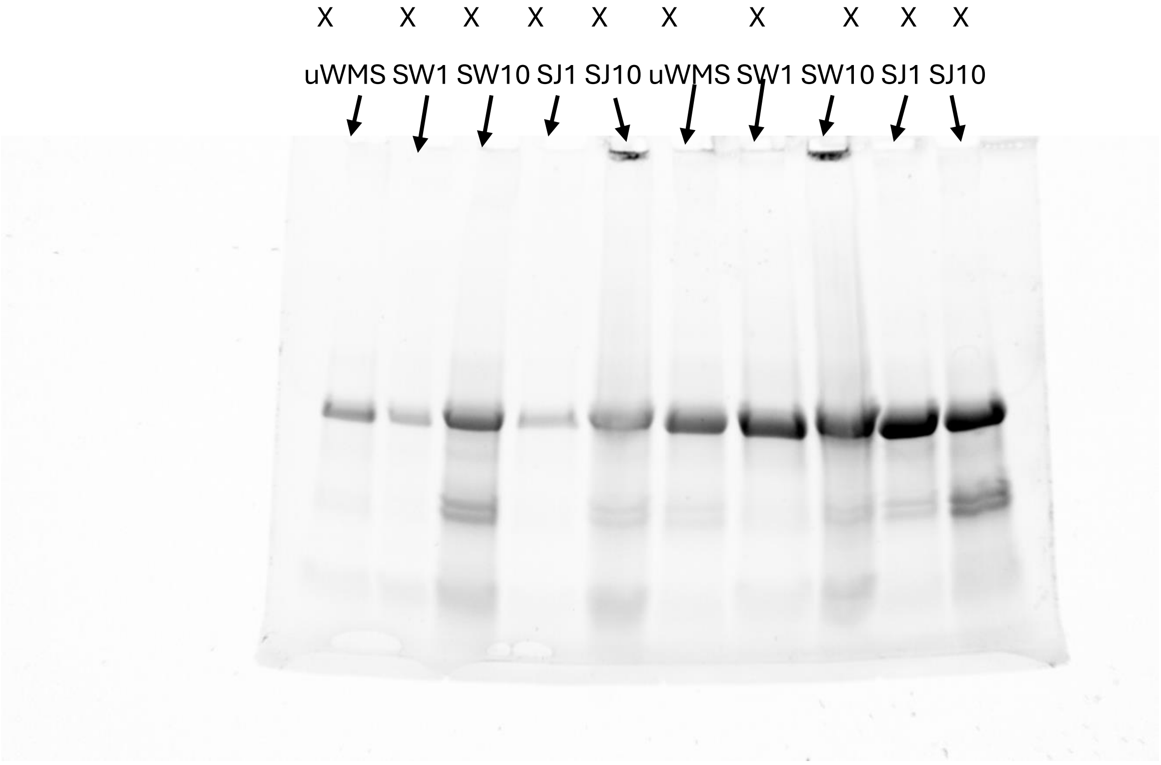

Figure 3:Participant 1 and 2 saliva samples on gel.

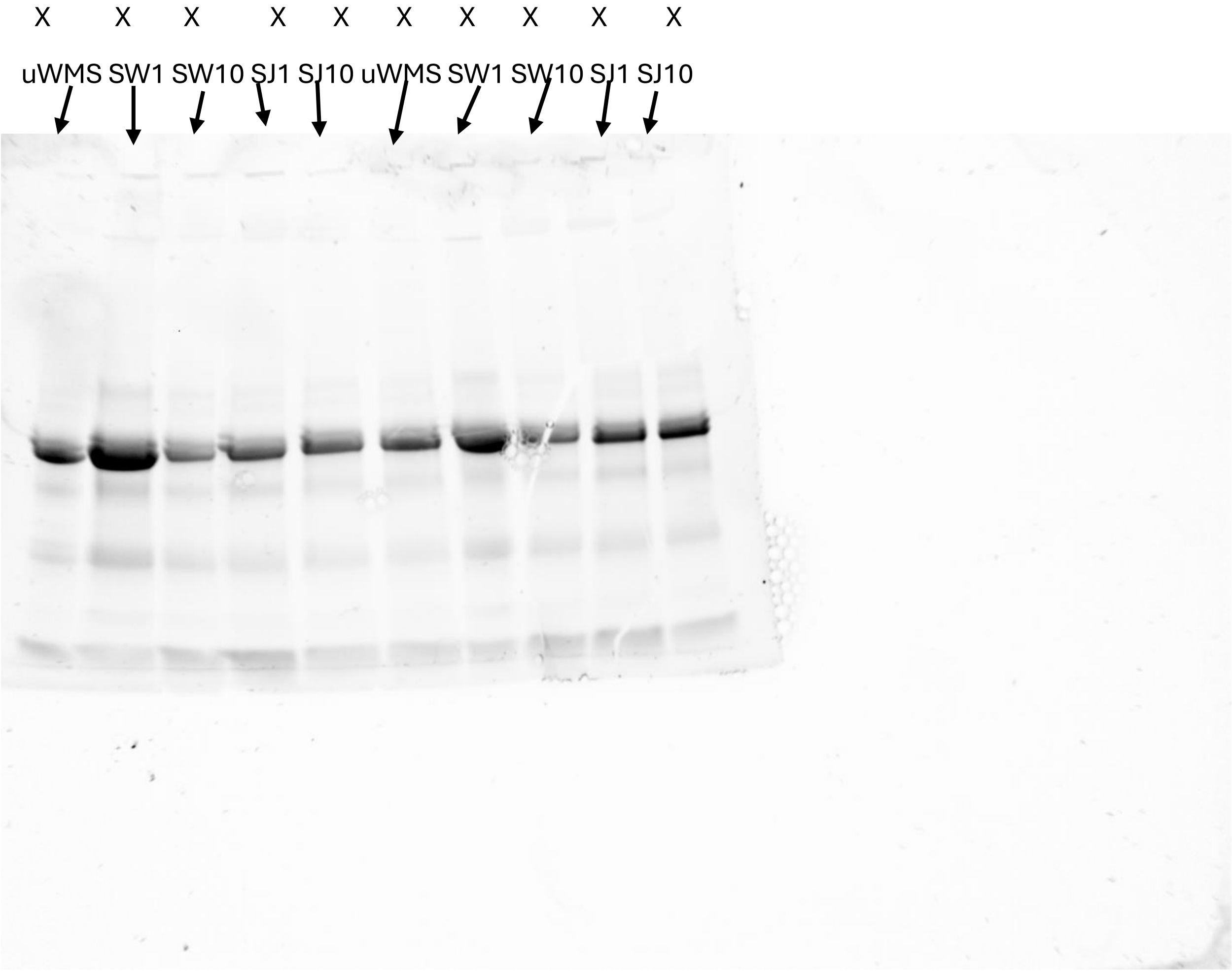

Figure 4 : Participant 3 and 4 saliva samples on a gel.

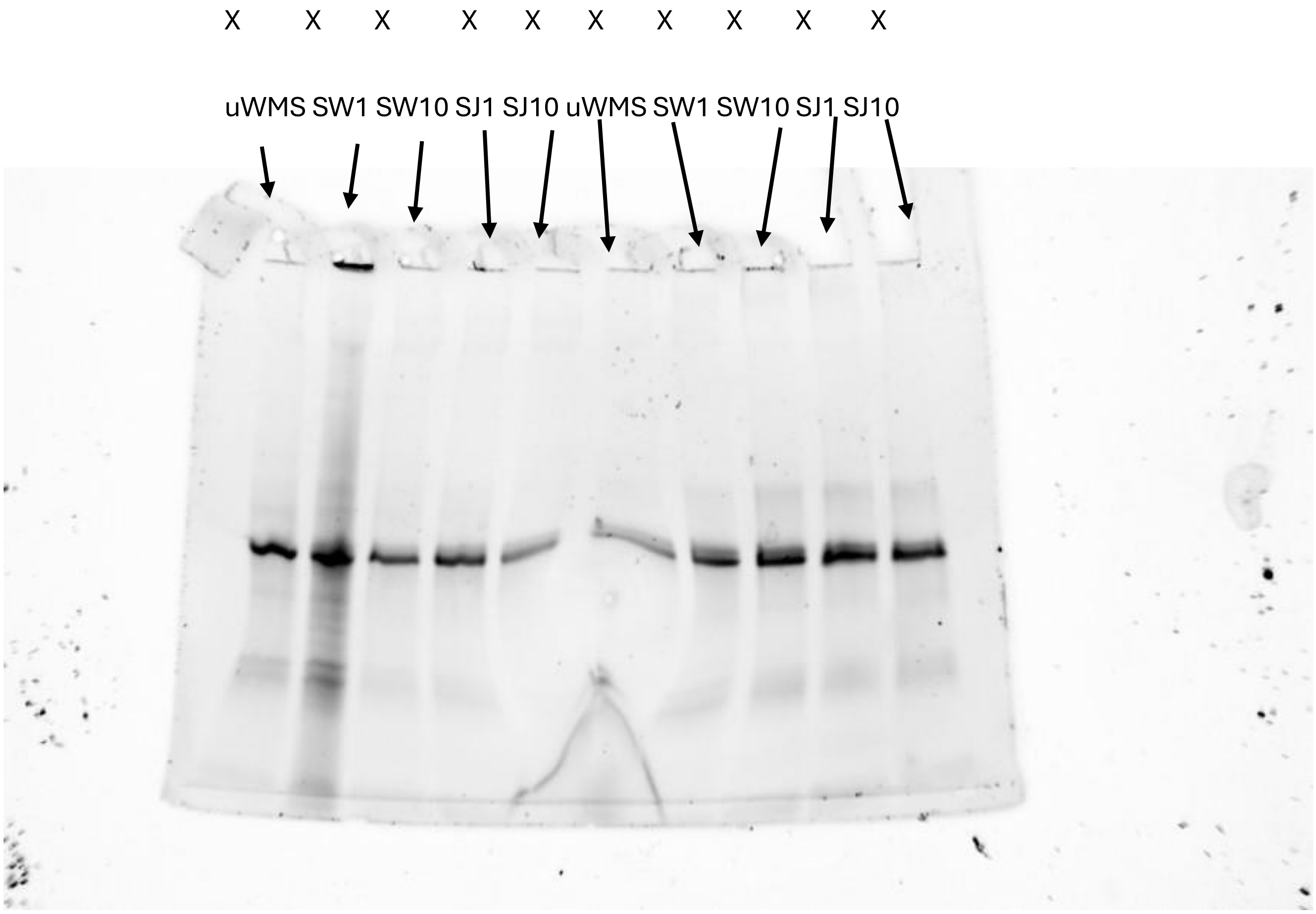

Figure 5: Participant 5 and 6 saliva samples on a gel.

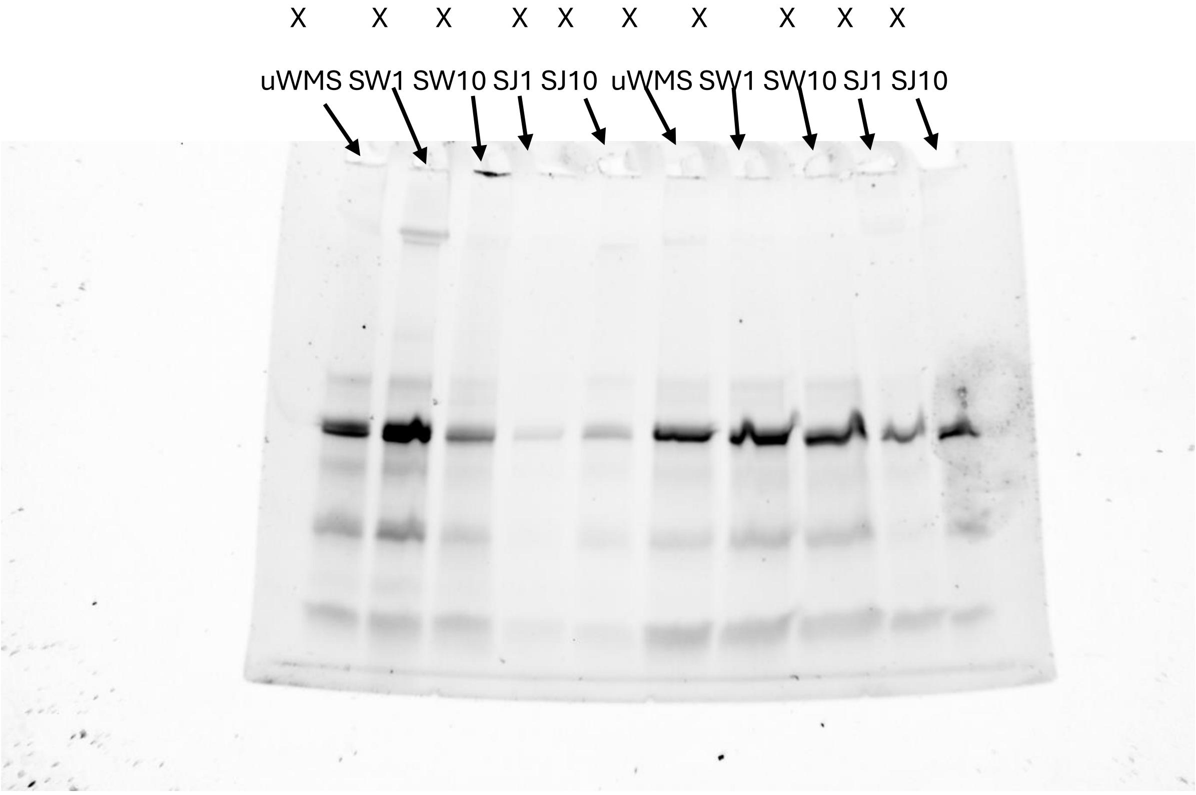

Figure 6: Participant 7 and 8 saliva samples

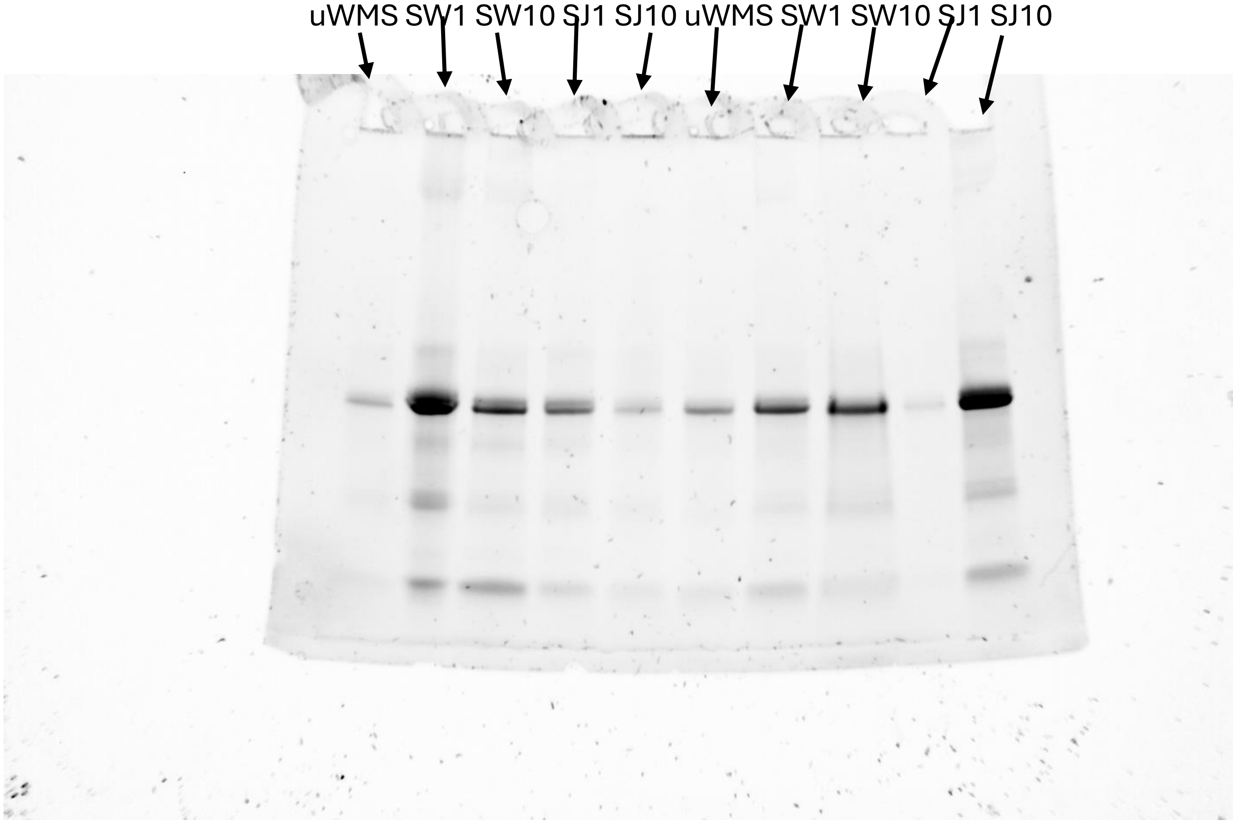

Figure7: Participant 9 and 10 saliva samples on a gel

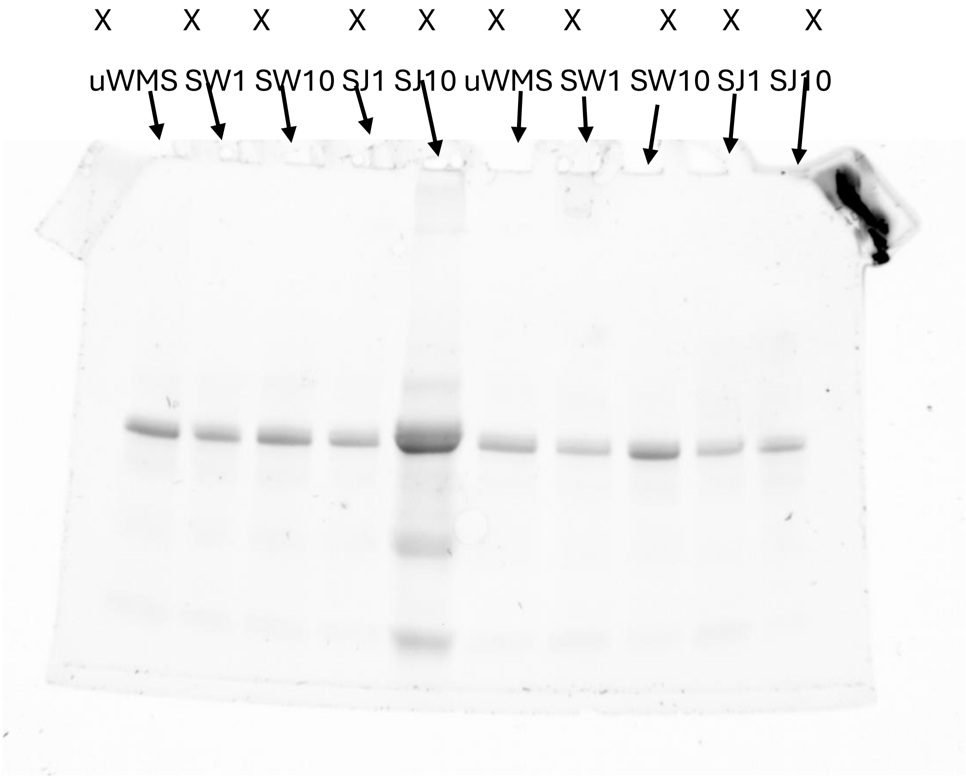

Figure8: Participant 11 and 12 saliva samples on a gel

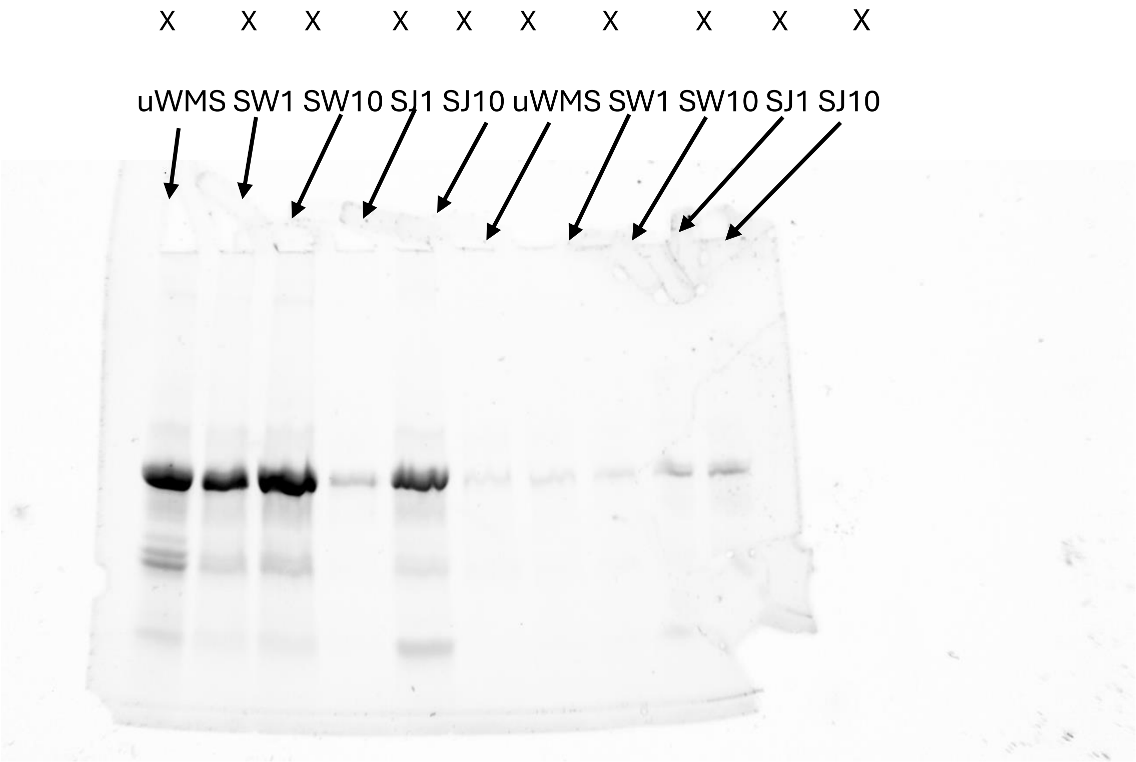

Figure 9: Participant 13 and 14 saliva samples on a gel

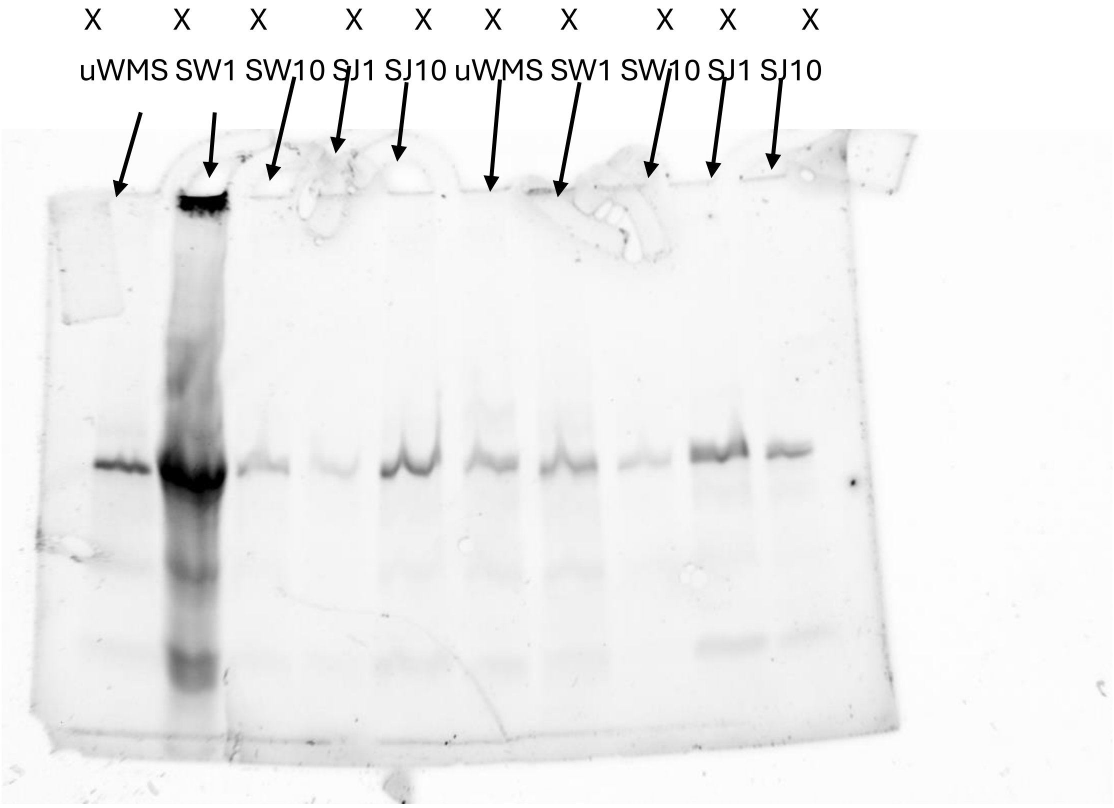

Figure 11: Participant 15 and 16 saliva samples on a gel.

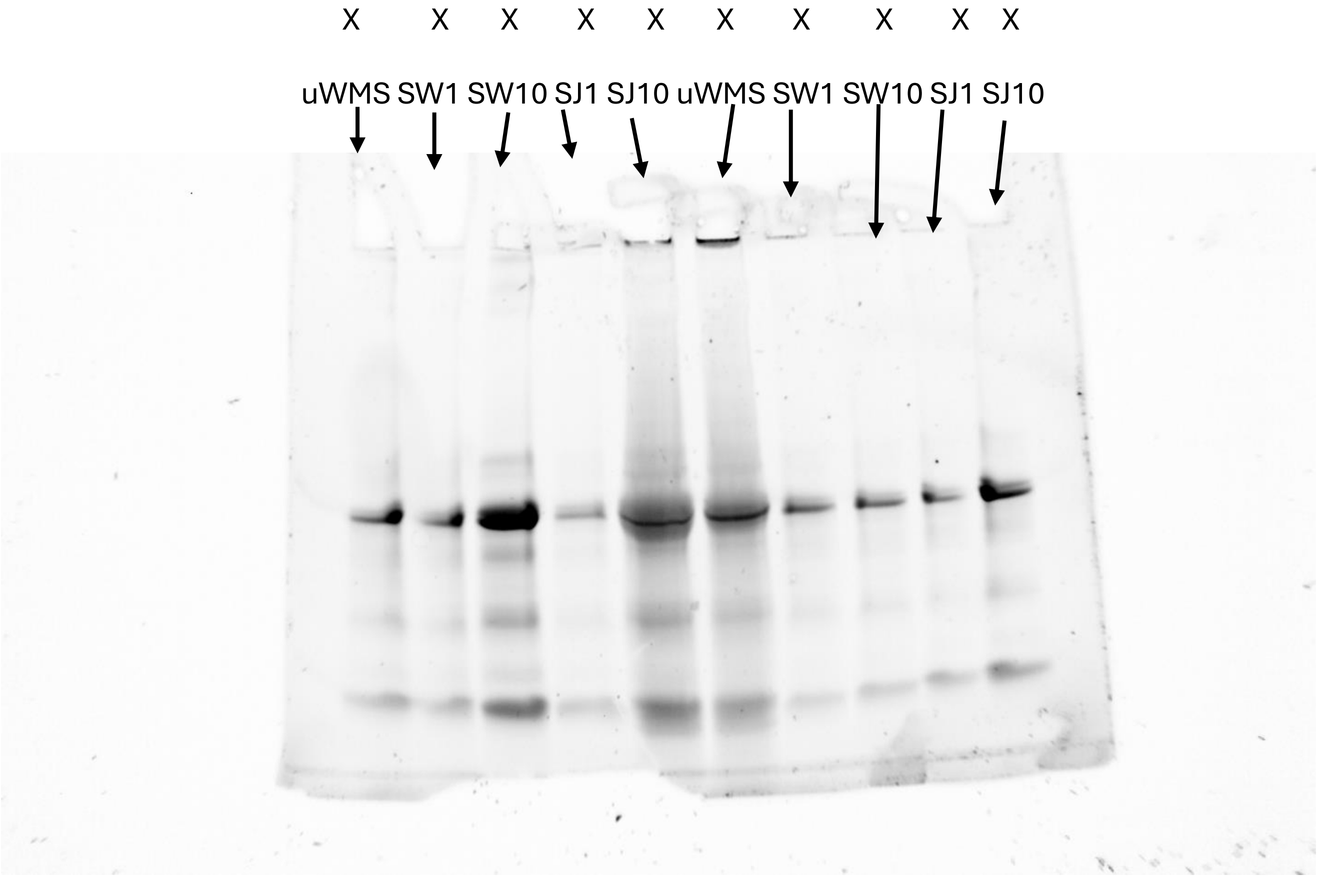

Figure12: Participant 17 and 18 saliva samples on a gel

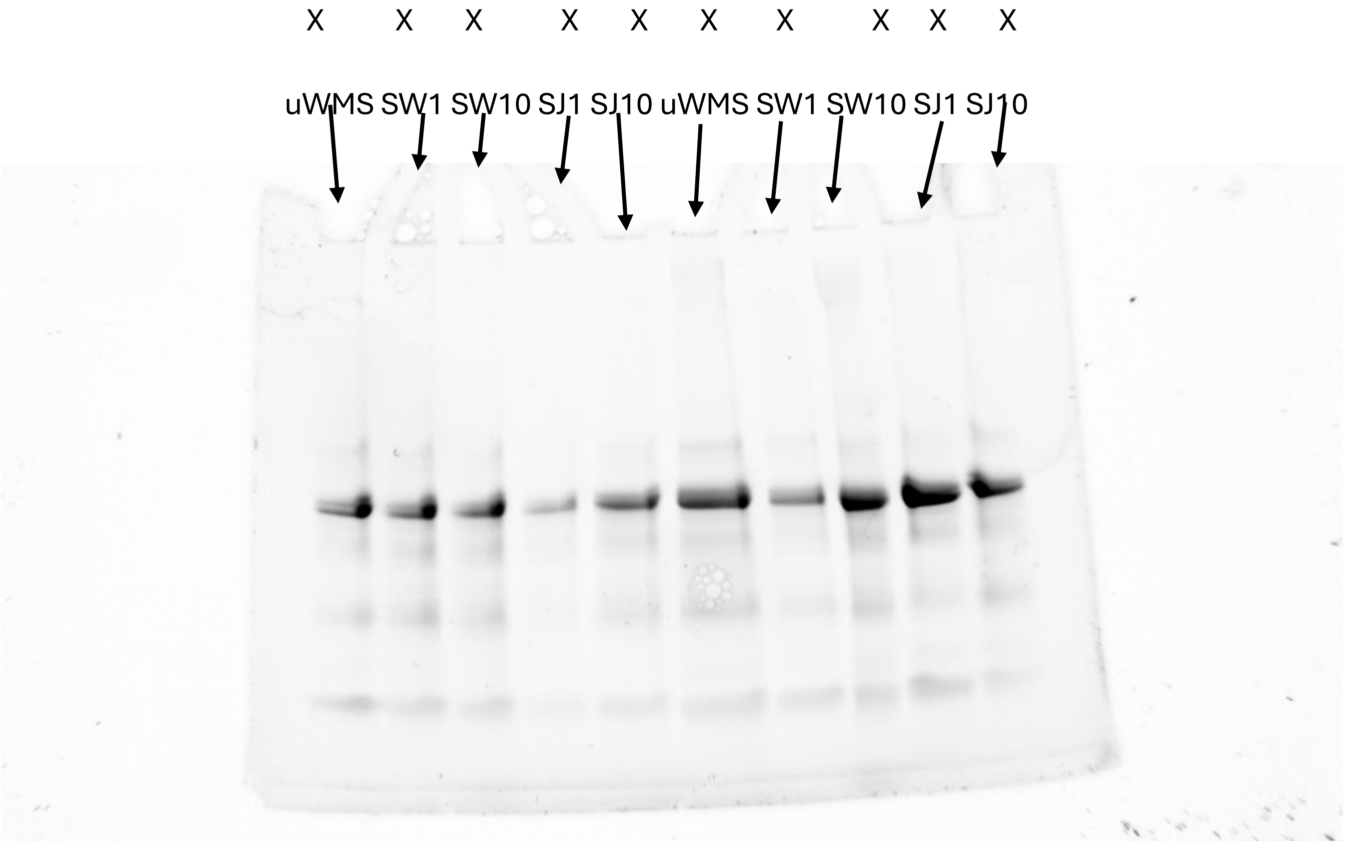

Figure13: Participant 19 and 20 saliva samples on a gel.
